# Dose-response relationship between cumulative physical workload and osteoarthritis of the hip – a meta-analysis applying an external reference population for exposure assignment

**DOI:** 10.1186/s12891-018-2085-8

**Published:** 2018-06-01

**Authors:** Andreas Seidler, Laura Lüben, Janice Hegewald, Ulrich Bolm-Audorff, Annekatrin Bergmann, Falk Liebers, Christina Ramdohr, Karla Romero Starke, Alice Freiberg, Susanne Unverzagt

**Affiliations:** 1 Institute and Policlinic of Occupational and Social Medicine (IPAS), Faculty of Medicine, Technical University Dresden, Fetscherstr. 74, 01307 Dresden, Germany; 2 Division of Occupational Health, Department of Occupational Safety and Environment, Regional Government of South Hesse, Wiesbaden, Germany; 3 Justus-Liebig-University, Gießen, Germany; 4 Institute for Medical Epidemiology, Biostatistics, and Informatics, Martin-Luther-University Halle-Wittenberg, Halle/Saale, Germany; 5 Federal Institute of Occupational Safety and Health, Berlin, Germany

**Keywords:** Physical workload, Osteoarthritis of the hip, Meta-regression, Exposure-risk relationship

## Abstract

**Background:**

There is consistent evidence from observational studies of an association between occupational lifting and carrying of heavy loads and the diagnosis of hip osteoarthritis. However, due to the heterogeneity of exposure estimates considered in single studies, a dose-response relationship between cumulative physical workload and hip osteoarthritis could not be determined so far.

**Methods:**

This study aimed to analyze the dose-response relationship between cumulative physical workload and hip osteoarthritis by replacing the exposure categories of the included studies with cumulative exposure values of an external reference population. Our meta-regression analysis was based on a recently conducted systematic review (Bergmann A, Bolm-Audorff U, Krone D, Seidler A, Liebers F, Haerting J, Freiberg A, Unverzagt S, Dtsch Arztebl Int 114:581–8, 2017). The main analysis of our meta-regression comprised six case-control studies for men and five for women. The population control subjects of a German multicentre case-control study (Seidler A, Bergmann A, Jäger M, Ellegast R, Ditchen D, Elsner G, Grifka J, Haerting J, Hofmann F, Linhardt O, Luttmann A, Michaelis M, Petereit-Haack G, Schumann B, Bolm-Audorff U, BMC Musculoskelet Disord 10:48, 2009) served as the reference population. Based on the sex-specific cumulative exposure percentiles of the reference population, we assigned exposure values to each category of the included studies using three different cumulative exposure parameters. To estimate the doubling dose (the amount of physical workload to double the risk of hip osteoarthritis) on the basis of all available case-control-studies, meta-regression analyses were conducted based on the linear association between exposure values of the reference population and the logarithm of reported odds ratios (ORs) from the included studies.

**Results:**

In men, the risk to develop hip osteoarthritis was increased by an OR of 1.98 (95% CI 1.20–3.29) per 10,000 tons of weights ≥20 kg handled, 2.08 (95% CI 1.22–3.53) per 10,000 tons handled > 10 times per day and 8.64 (95% CI 1.87–39.91) per 10^6^ operations. These estimations result in doubling dosages of 10,100 tons of weights ≥20 kg handled, 9500 tons ≥20 kg handled > 10 times per day and 321,400 operations of weights ≥20 kg. There was no linear association between manual handling of weights at work and risk to develop hip osteoarthritis in women.

**Conclusions:**

Under specific conditions, the application of an external reference population allows for the derivation of a dose-response relationship despite high exposure heterogeneities in the pooled studies.

**Electronic supplementary material:**

The online version of this article (10.1186/s12891-018-2085-8) contains supplementary material, which is available to authorized users.

## Background

There is consistent evidence from observational studies of an association between occupational lifting and carrying of heavy loads and the diagnosis of hip osteoarthritis (for the three systematic reviews published after 2010, see [9, 19] and [2]). However, due to the heterogeneity of exposure estimates (differing weights, frequencies and/or duration of manual handling considered in the single studies), a dose-response relationship between cumulative physical workload and hip osteoarthritis (OA) could not be determined so far. Sulsky et al. [19] concluded in their systematic review that it is “not possible to estimate a quantitative dose-response relationship between workload and hip OA using existing data of loads”. However, it would be important to derive the “doubling dose” of cumulative physical workload, as in many countries the recognition and compensation of occupational diseases is based on the “doubling risk” criterion which is usually equated with a probability of causation of 50% [14, 16].

In a recently published systematic review [2], we pooled the risk estimates for the highest categories of the included studies. For men, based on seven case-control studies [3, 5–7, 10, 12, 17, 26], we found a pooled odds ratio (OR) of 2.1 (95% CI 1.4–3.1) for the highest exposure categories combined. Although this OR almost perfectly reflects the targeted “doubling risk”, it was not possible to determine a concrete “doubling dose” from the included case-control studies. The underlying problems shall be illustrated using the study of Kaila-Kangas et al. [10] (Table 1), one of the best-rated studies in our systematic review.

**Table 1:** Hip osteoarthritis risks in males according to the study of Kaila-Kangas et al. [10]

Category	Duration of exposure	Manually handled weights	Frequency per shift	Percentage of participants in the corresponding category	Mean percentile of exposure	Risk estimate (OR)
1	0 yrs.	> 20 kg	≥ 10×	53.6%^b^	27th	1.0 -
2	1–12 yrs.^a^	> 20 kg	≥ 10×	16.1%^b^	62nd	1.1 (95% CI 0.4–3.2)
3	13–24 yrs.^a^	> 20 kg	≥ 10×	12.7%^b^	76th	2.2 (95% CI 0.8–5.9)
4	> 24 yrs.^a^	> 20 kg	≥ 10×	17.6%^b^	91st	2.3 (95% CI 1.2–4.3)

^a^one working year corresponds to 220 working days

^b^The authors only specify the case numbers in the single exposure categories; for the total participants, the numbers were obtained from the authors [11]. The mean percentiles of exposure are based on these percentages. To calculate the mean percentile of exposure, the cumulative percentage of the less exposed categories plus the halved percentage of the considered category were summed up. For example, the mean percentile of exposure for category 4 was calculated as the (53.6 + 16.1 + 12.7 + 17.6/2) = 91st percentile

In this case-control study, Kaila-Kangas et al. [10] found a monotonous risk increase with increasing duration of load handlings. However, a cumulative dose cannot be calculated for the individual exposure categories since greater than 10 load handlings per shift could represent an average of 11, 50 or even 100 load handlings per shift. Moreover, the parameters of exposure differ considerably from the exposure parameters of most of the other included studies: while in the aforementioned study of Kaila-Kangas et al. [10] as well as in the study of Croft et al. [5, 6] risk estimates are related to the duration of exposure, the other studies consider maximum loads [3], frequency of lifting or carrying of loads [7, 12], cumulatively lifted tons [26], or cumulative “ton-years” ([17]; one ton-year meaning one ton lifted per day for one year).

The basic idea of this meta-regression analysis was to uniformly replace the exposure categories of the included studies with cumulative exposure values using an external (German) reference population: if a risk estimate of an included study was related to a specific exposure percentile in the originally studied population, this risk estimate was then linked to the same exposure percentile of the external reference population. The control group of a German multi-centre population-based case-control study (“EPILIFT” study; [18]) was chosen as the reference population.

## Methods

### Systematic literature search

This meta-regression analysis was based on our recently published systematic review [2] on the relationship between physical workload and osteoarthritis of the hip. We first performed an update (until March 31, 2017) of our literature search using the published search strategy. The titles and abstracts of studies identified by the electronic database searches were screened independently by two reviewers. Afterwards, the full-texts of the remaining articles were screened by the two reviewers. As a result of this updated search, no further studies were identified for inclusion in our meta-regression analysis.

### Reference population

The population control subjects of a German multicentre case-control study [18] served as the reference population. The participants (453 men and 448 women) were selected randomly from a 1 % random sample of residents aged 25 to 70 years drawn by the local population registration offices of four study regions in Germany (Frankfurt/Main, Freiburg, Halle, Regensburg). To approximate the age distribution of the included studies, we restricted the reference population to individuals aged 40 years or more. The mean age of men was 54.4 years (median 55 years; range 40–71 years), and the mean age of women was 52.9 years (median 52 years; range 40–70 years).

In the reference study exposure assessment was based on expert evaluation [18]. Those subjects who, on the basis of self-reported information, exceeded relatively low “exposure thresholds”, received a semi-standardized comprehensive expert interview performed by occupational hygienists of the institutions for statutory accident insurance and prevention with special experience in the assessment of occupational load handling. Based on specific job task supplementary surveys, the occupational hygienists assessed the intensity, frequency and duration of specific spine-related exposures induced, inter alia, by manual handling of weights.

We calculated the exposure percentiles for the reference population, separately for men and women, for three different cumulative exposure parameters (see Table 2):

- cumulative weight [in tons] lifted and/or carried, taking into account all weights ≥20 kg;
- cumulative weight [in tons] lifted and/or carried, taking into account all weights ≥20 kg that were handled at least 10 times per working day;
- cumulative number of lifting and/or carrying operations of weights ≥20 kg.

**Table 2:** Cumulative exposure percentiles of the reference population ≥ 40 years

Assigned exposure parameters	Sex	10th	20th	30th	40th	50th	60th	70th	80th	90th	100th
a. cumulative tons of weights ≥20 kg handled	M	0	0	0	8	8	281	1054	2620	6101	307,813
	F	0	0	0	0	0	0	0	13	455	17,101
b. cumulative tons of weights ≥20 kg handled ≥10 times/day	M	0	0	0	0	0	116	784	2220	5971	307,813
	F	0	0	0	0	0	0	0	0	64	17,101
c. cumulative number [× 1000] of lifting and/or carrying operations of weights ≥20 kg	M	0	0	0	0.6	0.6	11	35	80	218	13,463
	F	0	0	0	0	0	0	0	0.2	18	732

### Exposure assignment to the single categories of the included studies

Based on the cumulative exposure percentiles of the reference population, we assigned exposure values to each individual exposure category of the included studies. This procedure shall be explained again taking the Kaila-Kangas et al. [10] study as an example: in this study, the mean percentile of exposure for the reference category (category 1) was the 27th percentile (see Table 1). For the 27th percentile of the reference population, all exposures were zero (see Table 2); we therefore assigned zero-exposures to the reference category. The mean percentile of the category 2 was the 62nd percentile (Table 1). The 62nd percentile of the reference population meant an exposure of 353 tons (exposure a), 194 tons (exposure b), and 12,000 lifting and/or carrying operations (exposure c). Hence, these exposure values were assigned to the category 2 of the Kaila-Kangas et al. [10] study (see Table 3, columns “assigned exposure”). For categories 3 and 4 of the mentioned study, we proceeded accordingly. In Tables 3 (men) and 4 (women), the crude as well as the age-corrected (for men in studies with a mean age of 60 years or more; see Additional file 1: Table S1) cumulative exposures assigned to each exposure category of the included studies are presented (Table 4).

**Table 3:** Cumulative exposure assignments among men for the single exposure categories

Study, country	Median age (ref. population: 47.3 years, range 25–70 yrs.)	Exposure parameter of the included study (not used for analysis)	Cases, n	Control subjects/Participants^a^, n (%)	Median exposure percentile	OR (95% CI)	Assigned exposure parameter a^b^ [tons]	Assigned exposure parameter b^c^ [tons]	Assigned exposure parameter c^d^ [numbers × 1000]	Assigned age-corrected^e^exposure a. [tons]	Assigned age-corrected^e^exposure b. [tons]	Assigned age-corrected^e^exposure c. [numbers × 1000]
Coggon et al. [3], GB	mean 68 years (≥ 45 years)^f^	Duration of lifting loads ≥25 kg	210 (total)	210 (total)								
		0 years	91	115 (54.8%)	27.4	1.0 -	0	0	0	0	0	0
		1–9 years	22	28 (13.3%)	61.5	0.8 (0.4–1.7)	353	194	12	615	246	15
		10–19 years	14	15 (7,1%)	71.7	1.5 (0.6–3.8)	1299	968	41	1331	1054	46
		≥ 20 years	83	52 (24.8%)	87.6	2.3 (1.3–4.4)	4632	4354	170	5505	5168	191
Croft et al. [5, 6], GB	range 60–75 years	Duration of lifting or moving weights > 25.4 kg by hand	49 (total)	262^g^ (total)								
		< 1 years	9	71 (27.1%)	13.6	1.0 -	0	0	0	0	0	0
		1–19 years	14	106 (40.5%)	47.4	1.2 (0.5–2.9)	8	0	0.6	8	0	0.3
		≥ 20 years	26	85 (32.4%)	83.8	2.5 (1.1–5.7)	3380	3252	107	4449	3818	124
Elsner et al. [7], Germany	ca. 51 years (43% ≤45 yrs.)	Lifting > 20 kg frequently or almost always	134 (total)	95 (total)								
		No	73	61 (64.2%)	32.1	1.0 -	0	0	0	0	0	0
		Yes	61	34 (35.8%)	82.1	1.1 (0.65–2.10)	3061	2670	88	3061	2670	88
Kaila-Kangas et al. [10], Finland	ca. 51 years (range 30–97 years)	Duration of manual handling of loads > 20 kg	59 (total)	2,853^h^ (total)								
		0 years	19	1,561^i^ (53.6%)	26.8	1.0 -	0	0	0	0	0	0
		1–12 years	7	468^i^ (16.1%)	61.7	1.1 (0.4–3.2)	353	194	12	353	194	12
		13–24 years	10	371^i^ (12.7%)	76.1	2.2 (0.8–5.9)	1725	1428	65	1725	1428	65
		> 24 years	23	514^i^ (17.6%)	91.2	2.3 (1.2–4.3)	6687	6432	278	6687	6432	278
Lau et al. [12], Hong Kong	Not reported	Lifting ≥50 kg	30 (total)	90 (total)								
		No	17	80 (88.9%)	44.5	1.0 -	0	0	0.6	0	0	0
		1–10 times per week	5	4 (4.4%)	91.1	8.5 (1.6–45.3)	6687	6432	278	10,866	9076	336
		≥10 times per week	8	6 (6.7%)	96.7	9.6 (2.2–42.2)	21,837	21,823	607	24,695	24,278	727
Rubak et al. [17], Denmark	64.3 years (range ca. 40–70 yrs.)	No. of ton years (1 ton year = lifting 1 ton per day over 1 year)	957 (total)	1759 (total)								
		0 ton years	390	779	22.2	1.0 -	0	0	0	0	0	0
		> 0- < 10 ton years	164	333	53.8	1.0 (0.8–1.3)	92	0	26	116	0	5
		10- < 20 ton years	153	304	71.9	0.9 (0.7–1.2)	1299	968	41	1331	1054	46
		20–115 ton years	250	343	90.3	1.4 (1.1–1.7)	6527	5980	219	8946	6668	325
Vingard et al. [26], Sweden	63 years (range 50–70 yrs.)	Lifted tons before the age of 49	233 (total)	302 (total)								
		0–137 tons		60%^j^	30.0	1.0 -	0	0	0	0	0	0
		138–3006 tons		20%^j^	70.0	1.6 (0.9–2.7)	1054	784	34	1226	968	41
		3007–94,003 tons		20%^j^	90.0	1.8 (1.1–3.0)	6101	5971	218	7177	6596	280

^a^In the study of Kaila-Kangas et al. [10], the distribution of participants is given

^b^Cumulative weight [in tons] lifted and/or carried, taking into account all weights ≥20 kg

^c^Cumulative weight [in tons] lifted and/or carried, taking into account all weights ≥20 kg that were handled at least 10 times per working day

^d^Cumulative number of lifting and/or carrying operations of weights ≥20 kg

^e^For studies with a mean age > 60, the reference population is restricted to individuals ≥50 years

^f^According to Cooper et al. [4]

^g^Excluding 32 control subjects with missing values

^h^Excluding 257 participants with missing values

^i^Kaila-Kangas L. Personal communication. Email dated April 3, 2017

^j^Vingard et al. [26] do not give the numbers of cases and control subjects in the single exposure categories. Exposure categories were formed as follows: “*Three exposure groups were defined on the basis of the loads in the reference group. Those unexposed and the 5% less exposed were considered to have low exposure. The rest of the exposed group was divided into two equally large groups, classified as the medium-exposure group and the high-exposure group*” ([26], p. 106). In the Vingard et al. [26] study, the reference category includes all individuals with an exposure of up to 137 tons. In our reference population, a 137 ton-exposure corresponds to the 60th exposure percentile. We therefore assigned the 30th mean exposure percentile to the reference category

**Table 4:** Cumulative exposure assignments among women for the single exposure categories

Study	Median age (ref. population: 46 years, range 25–70 yrs.)	Exposure parameter of the included study	Cases, n	Control subjects/participants^a^, n (%)	Median exposure percentile	OR (95% CI)	Assigned exposure a^b^[tons]	Assigned exposure b^c^ [tons]	Assigned exposure c^d^[numbers × 1000]
Coggon et al. [3], GB	mean 68 years (≥ 45 years^e^)	Duration of lifting loads ≥25 kg	401 (total)	401 (total)					
		0 years	328	334 (83.3%)	41.7	1.0 -	0	0	0
		1–9 years	40	35 (8.7%)	87.7	1.1 (0.6–1.7)	238	0	12
		10–19 years	19	12 (3%)	93.5	1.4 (0.7–2.9)	1124	575	45
		≥ 20 years	14	20 (5%)	97.5	0.8 (0.4–1.5)	4576	3468	176
Elsner et al. [7], Germany	ca. 49 years (54% ≤45 yrs.)	Lifting > 20 kg frequently or almost always	86 (total)	103 (total)					
		no	68	92 (89.3%)	44,7	1.0 -	0	0	0
		yes	18	11 (10.7%)	94,7	1.9 (0.83–4.79)	1522	1332	52
Kaila-Kangas et al. [10], Finland	ca. 53 years (range 30–97 years)	Duration of manual handling of loads > 20 kg	71 (total)	3,430^f^ (total)					
		0 years	40	2687 (76.8%)	38.4	1.0 -	0	0	0
		1–12 years	7	312 (8.9%)	81.2	1.6 (0.7–3.5)	13	0	0.7
		13–24 years	8	193 (5.5%)	88.4	3.8 (1.7–8.1)	288	0	14
		> 24 Jahre	16	306 (8.7%)	95.7	1.2 (0.7–2.1)	1949	1794	63
Lau et al. [12], Hong Kong		Lifting ≥50 kg	108 (total)	324 (total)					
		No	77	277 (85.5%)	42.8	1.0 -	0	0	0
		1–10 times per week	10	18 (5.6%)	88.3	2.0 (0.9–4.6)	288	0	14
		≥10 times per week	21	29 (9.0%)	95.6	2.9 (1.5–5.6)	1949	1794	63
Rubak et al. [17], Denmark	64.7 years (range ca. 40–70 yrs.)	No. of ton years (1 ton year = lifting 1 ton per day over 1 year)	935^g^ (total)	1721 (total)					
		0 ton years	527	1000 (58.1%)	29.1	1.0 -	0	0	0
		> 0- < 10 ton years	169	267 (15,5%)	66	1.15 (0.87–1.53)	0	0	0
		10- < 20 ton years	136	288 (16,7%)	82.1	0.81 (0.61–1.09)	24	0	0
		20–115 ton years	103	166 (9,6%)	95.2	1.0 (0.72–1.41)	1546	70	19
Vingard et al. [25], Sweden	63 years (range 50–70 yrs.)	Number of lifts before the age of 50	230 (total)	273 (total)					
		0–20,328	47	68 (25%)	12.5	1.0 -	0	0	0
		20,329–44,088	101	137 (50%)	50	1.1 (0.7–1.7)	0	0	0
		44,089–95,040	82	68 (25%)	87.6	1.5 (0.9–2.5)	194	0	12

^a^In the study of Kaila-Kangas et al. [10], the distribution of participants is given

^b^Cumulative weight [in tons] lifted and/or carried, taking into account all weights ≥20 kg

^c^Cumulative weight [in tons] lifted and/or carried, taking into account all weights ≥20 kg that were handled at least 10 times per working day

^d^Cumulative number of lifting and/or carrying operations of weights ≥20 kg

^e^According to Cooper et al. [4]

^f^Excluding 16 participants with missing values

^g^Excluding 4 cases with missing values

In the core analyses, we excluded the study by Lau et al. [12], because the distribution of occupations and the working conditions might differ considerably between Hong Kong and Europe.

To estimate the doubling dose, we examined the linear relationship between the assigned cumulative exposure values and the log ORs of the osteoarthritis risk by conducting random-effects meta-regressions using mixed models with the inverse-distance weighted method (SAS v. 9.2 proc. mixed). We regarded the separate exposure categories as separate observations and clustered them by their corresponding studies. Moreover, we conducted a first sensitivity analysis, in which we based the estimation of the doubling dose on the 90th percentile of the cumulative exposure values of the reference population. We based this analysis on the meta-analysis by [2], who found a relative risk of about 2 when pooling the highest categories of the included studies. In a second sensitivity analysis, we included the study conducted by Lau et al. [12]. In a third sensitivity analysis, we assigned the age-corrected exposure values for men (see Table 3) to studies with a mean age of 60 years or more.

## Results

**Fig. 1:**
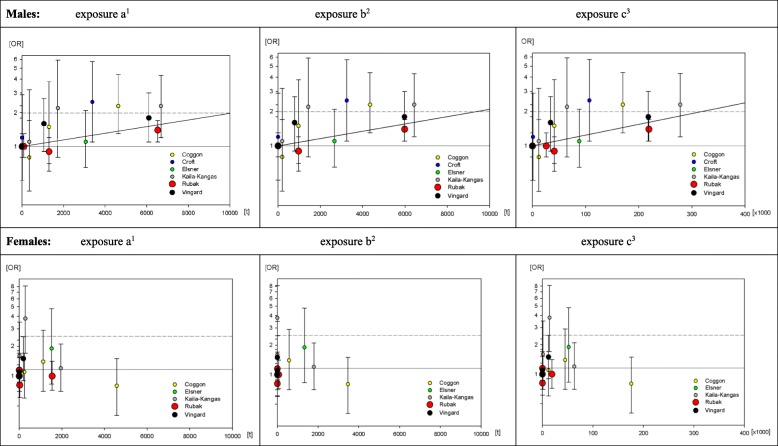
Risk estimates among males and females of the included studies for a. cumulative tons of weights ≥20 kg handled, b. cumulative tons of weights ≥20 kg handled > 10 times / day, c. cumulative lifting and / or carrying operations of weights ≥20 kg and pooled risk increase (grey line). Legend: the circle size reflects the large [17], medium [26] and small [3, 5–7, 10] weights of the included studies in the meta-regression analysis. ^1^ Cumulative weight [in tons] lifted and/or carried, taking into account all weights ≥20 kg. ^2^ Cumulative weight [in tons] lifted and/or carried, taking into account all weights ≥20 kg that were handled at least 10 times per working day. ^3^ Cumulative number of lifting and/or carrying operations of weights ≥20 kg

Risk estimates increased with increasing cumulative exposure among men in all studies included. Figure 1 summarizes the exposures and resulting risk estimates for men and women.

Our meta-regression analysis, based on six European studies [3, 5–7, 10, 17, 26], revealed a mean risk increase of 1.98 per 10,000 cumulative tons of weights ≥20 kg handled (95% CI 1.20–3.29) among men. This increase resulted in a doubling dose of 10,100 cumulative tons. When solely weights ≥20 kg handled > 10 times per day were taken into account, the doubling dose was 9500 cumulative tons. For cumulative lifting and/or carrying operations of weights ≥20 kg, we found a doubling dose of about 321,400 operations (Table 5).

**Table 5:** Doubling doses based on the pooled analyses for men, core analysis

	Core analysis^a^	
Assigned exposure parameters	Pooled OR (95% CI)	Doubling dose^b^
a. cumulative tons of weights ≥20 kg handled	1.98 per 10,000 tons (95% CI 1.20–3.29)	10,100 tons (95% CI 5800–38,800 tons)
b. cumulative tons of weights ≥20 kg handled > 10 times/day	2.08 per 10,000 tons (95% CI 1.22–3.53)	9500 tons (95% CI 5500–34,900 tons)
c. cumulative number [× 1000] of lifting and/or carrying operations of weights ≥20 kg	8.64 per 10^6^ handlings (95% CI 1.87–39.91)	321,400 (95% CI 188,000–1,106,600)

^a^ For all studies, the reference population includes all individuals ≥40 years

^b^ calculated with the exact pooled OR and rounded down to hundred

Among women (Fig. 1, lower half), only three out of the six eligible studies found increased risk estimates with increasing cumulative exposures [7, 12, 25]. Two studies with less than 5% of women in the highest exposure category found increased risks in the lower exposure categories, followed by a decreased risk in the highest exposure category [3, 10]. The study of Rubak et al. [17] found no positive relationship between cumulative exposure and hip osteoarthritis among women. In the meta-regression, there was no statistically significant risk increase among women for any of the examined cumulative exposure parameters. We therefore did not conduct any further sensitivity analyses for women.

The following three sensitivity analyses were conducted for men:

- *Approximating the doubling dose by the 90th cumulative exposure percentile (sensitivity analysis 1)*

For all studies (except for the small study of [7] with only two exposure categories) we assigned the highest exposure category among men at approximately the 90th exposure percentile (range of percentiles of the highest exposure category: 82.1st to 91.2nd; median: 90.2nd percentile). The previous pooled analysis (see [2]) found that the highest exposure categories of the studies included approximately doubled the risk among men (OR 2.09; 95% CI 1.4–3.1). Therefore, the doubling dose should correspond to approximately the 90th percentile of the cumulative exposure values of the reference population. Among men, the 90th cumulative exposure percentiles of our reference population are (see Tables 2 and 6, sensitivity analysis 1):

- 6100 cumulative tons of weights ≥20 kg handled;
- 6000 cumulative tons of weights ≥20 kg handled > 10 times / day;
- 218,000 cumulative lifting and / or carrying operations of weights ≥20 kg.

**Table 6:** Sensitivity analyses: doubling doses based on the pooled analyses

Assigned exposure parameters	Sensitivity analysis	Pooled OR (95% CI)	Doubling dose^a^
a. cumulative tons of weights ≥20 kg handled	1	2.09 (95% CI 1.40–3.10) for appr. the 90th percentile of the studies included by Bergmann et al. [2]	6100 tons
	2	2.15 per 10,000 tons (95% CI 1.39–3.34)	9000 tons
	3	1.64 per 10,000 tons (95% CI 1.13–2.40)	14,000 tons
b. cumulative tons of weights ≥20 kg handled > 10 times/day	1	2.09 (95% CI 1.40–3.10) for appr. the 90th percentile of the studies included by Bergmann et al. [2]	6000 tons
	2	2.24 per 10,000 tons (95% CI 1.42–3.54)	8600 tons
	3	1.94 per 10,000 tons (95% CI 1.20–3.14)	10,500 tons
c. cumulative number [× 1000] of lifting and/or carrying operations of weights ≥20 kg	1	2.09 (95% CI 1.40–3.10) for appr. the 90th percentile of the studies included by Bergmann et al. [2]	218,000
	2	11.86 per 10^6^ handlings (95% CI 2.99–47.06)	280,300
	3	3.85 per 10^6^ handlings (95% CI 1.39–10.70)	514,000

^a^ calculated with the exact pooled OR and rounded down to hundred

The advantage of this sensitivity analysis is that it abstains from the assumption of a linear dose-response relationship between cumulative exposure and hip osteoarthritis risk.

- *Analysis including Lau et al.* [12] *(sensitivity analysis 2)*

When the Hong Kong study of Lau et al. [12] was included, the risk estimators slightly increased (and, correspondingly, the doubling doses decreased) in men. We found a risk increase of 2.15 per 10,000 cumulative tons of weights ≥20 kg handled (95% 1.39–3.34), resulting in a doubling dose of 9000 cumulative tons (Table 6, analysis 2). When solely weights ≥20 kg were taken into account that were handled > 10 times per day, the doubling dose was 8600 cumulative tons. Considering cumulative lifting and/or carrying operations of weights ≥20 kg, we found a doubling dose of about 280,300.

- *Age-corrected meta-analysis (sensitivity analysis 3)*

When in an “age-corrected analysis” the reference population was restricted to individuals ≥50 years in studies with a mean age > 60 [3, 5, 6, 17, 26] (leaving the reference population of the other included studies unchanged), the risk estimators decreased (Table 6, analysis 3). This led to an increase of the doubling doses. The doubling dose was 14,000 tons for cumulative tons of weights ≥20 kg handled, 10,500 tons for cumulative tons of weights ≥20 kg handled more than 10 times per day, and about 514,000 for the cumulative number of lifting and/or carrying operations of weights ≥20 kg.

## Discussion

We developed a meta-regression approach to derive a dose-response relationship despite high heterogeneities of exposure assessments in the included primary studies. The basic idea of this approach was to uniformly replace the exposure categories of the included studies using cumulative exposure values from an external reference population. With this method, we estimated the exposure to lifting and/or carrying loads that resulted in a “doubling risk” of hip osteoarthritis (the “doubling dose”). We found a doubling dose in men between 6100 and 14,000 cumulative tons of weights ≥20 kg handled (exposure a.); between 6000 and 10,500 cumulative tons of weights ≥20 kg handled > 10 times/day (exposure b.); and between 218,000 and 514,000 cumulative lifting and/or carrying operations of weights ≥20 kg (exposure c.). The range of the estimated doubling dose might be particularly wide for the cumulative number of lifting and carrying operations ≥20 kg (exposure c.) since, unlike the other two exposure parameters, this cumulative measure does not take the weight of the single loads into account. Assuming a working life of 40 years and a working-year comprised of 220 days, the workload needed to achieve the doubling risk would be equivalent to either lifting 0.7 to 1.6 tons (exposure a.) resp. 0.7 to 1.2 tons (exposure b.) per day or performing between 25 to58 lifting and/or carrying operations of weights ≥20 kg (exposure c.).

There are some limitations of this newly developed approach:

1. *Comparability of the exposure distribution between studies*

As a basic assumption, the exposure distribution of manual handling of loads should be comparable between the study regions of the included studies. If the population-related amount of manual work were lower in the included studies than in our reference study, hip osteoarthritis risks at given exposure levels would tend to be underestimated. We therefore excluded Lau et al. [12], as the labour market differs considerably between Hong Kong (due to its large service sector and, for example, very small agricultural sector) and the other studies. However, the remaining European studies might also differ with respect to the distribution of occupational exposures. The German reference population was acquired between 2003 and 2005 in four regions which included rural areas, one large city (Frankfurt am Main, about 700.000 inhabitants), and three smaller cities (Regensburg, Halle, Freiburg, between 100.000 and 250.000 inhabitants). Two of the studies included in the meta-analysis were based on nationally representative population samples of Finland 2000–2001 [10] and Denmark 2009 [17]. Two studies were conducted in Great Britain: Coggon et al. ([3]; data collection 1993-95 in Portsmouth and North Staffordshire) and Croft et al. ([5, 6]; data collection 1982–1987 in North Staffordshire and Shrewsbury). According to labour market statistics (https://www.nomisweb.co.uk/reports/lmp/lep/1925185562/report.aspx?#ld), manufacturing might be slightly overrepresented in the North Staffordshire population relative to Great Britain in total. The study by Vingard et al. [26] was conducted in 1984–88 based on the referral areas of four Stockholm hospitals. According to the authors, the Greater Stockholm area where the study was performed was somewhat more urbanized than the rest of the country (Olsen et al. [15]). Elsner et al. [7] recruited control subjects in Frankfurt am Main (1989–93), and therefore service occupations might be overrepresented. Altogether, there are some differences in the distribution of occupations between the included studies, as well as between the included studies and the reference population. However, there is no indication of a severe over- or underrepresentation of heavy physical work in the included studies compared to the reference study.

Besides regional differences in the distribution of occupational exposure, time-effects must be taken into account. The included studies were conducted up to two decades [5, 6] earlier than our reference study. Because in earlier years a higher proportion of men had to fulfill physically highly demanding work, the substitution of exposures of earlier studies by exposures of a more recently recruited reference population might have overestimated the hip osteoarthritis risk at a given exposure level.

Moreover, the consequences of potential selection bias have to be taken into account. The response in the reference population (53% among control subjects, [18]) was lower than the response in all of the included studies (between 58% in [3] and 89% in [10]). As the proportion of blue-collar workers can be assumed to be higher among non-participating subjects [18], the relatively low response in the reference study might have led to an underestimation of physical workload. As a consequence, hip osteoarthritis risks at given exposure levels would tend to be overestimated.

Since several previous studies have consistently reported increased hip osteoarthritis risk among farmers [1, 5, 6, 8, 20–24], we intended to compare the proportion of agricultural occupations in the included studies and in the reference study. However, only two of the included studies give the occupations of the study subjects: among control subjects, the proportion of agricultural occupations was 15% in Croft et al. ([5, 6]; farmers and agricultural workers for at least one year) and 3% in Elsner et al. [7]. In our reference study, 7% of the control subjects had ever worked for at least half a year as agricultural, animal husbandry, or forestry worker [13]. This example points to potentially considerable between-study differences in the occupations of the study subjects.

2. *Potential age-dependency of cumulative occupational workload*

Up to the age of retirement, the cumulative exposure to manual handling of loads is expected to increase. We therefore roughly took the age distribution of the included studies into account by restricting the reference population to individuals aged 50 years or more for studies with a mean age of 60 years or more. As a result of this “age-corrected” sensitivity analysis, the doubling dose increased. A more precise consideration of potential age-effects would be possible 1) if the exact age distribution of the included studies was known and, 2) if a much larger reference population was available to allow for the accurate modeling of the age-distribution of the included studies.

3. *Questionable linearity of the dose-response relationship*

Our meta-analyses only examined linear models. However, there might be a threshold below which there is no risk increase of hip osteoarthritis. To also examine non-linear dose-response relationships, we intended to compare linear models with more complex (third-degree polynomial) models in a sensitivity analysis. However, according to a preliminary analysis, − presumably because of the low number of included studies and exposure categories – these complex models proved to be instable. In contrast, our first sensitivity analysis is independent of the linearity assumption, as only the relative risk around the 90th percentile is taken into account. Since we found lower doubling risks with this first sensitivity analysis, the assumption of a linear dose-response relationship might tend to overestimate the doubling dose. The (not yet proven) existence of an “effect threshold” might also (at least partly) explain our null findings among women. In comparison to men, the cumulative exposure of women is much lower. For example, the 90th exposure percentile of women for cumulative tons of weights ≥20 kg handled is only 7% of the 90th exposure percentile of men and might fall beyond a potential “effect threshold”. Limited power is an alternative (or additional) explanation for the null findings among women.

4. *Exposure uncertainties around the doubling dose*

According to our results, the doubling dose lies in a dose range in which small exposure differences are related to large risk changes. For example, among men, the 91st exposure percentile for cumulative tons of weights ≥20 kg handled is 21% higher than the 89th exposure percentile (6687 vs. 5505 tons). These uncertainties intensify with further increasing exposure: the 96st exposure percentile for cumulative tons of weights ≥20 kg handled is 54% higher than the 94th exposure percentile (19,993 vs. 12,983 tons). As a consequence, relatively small uncertainties in the assigned cumulative exposure might lead to large uncertainties of the doubling dose. Such uncertainties in the assigned cumulative exposure might not only result from regional differences in occupational workload (see under 1.) and from the age-dependency of cumulative physical workload (see under 2.), but also from exposure misclassification in the included single studies: exposure data was mostly based on participant self-reports, and not on objective measurements, expert ratings, or judgements. It is therefore possible that there was bias in the assignment of exposure categories, and such bias could have finally led to inaccurate percentile values.

Finally, we would like to point out that biased risk estimates in the included studies would also have led to biased pooled risk estimates in the meta-analysis. Case-control studies are particularly prone to recall bias. According to our quality assessment based on the Newcastle-Ottawa Assessment Scale and the Cochrane Handbook, the quality of the six case-control studies included in the meta-analysis was rather good (attaining 5 to 14 of 15 points, median 12.5 points; see [2]). However, five of the six case-control studies were based on self-reported exposure information. We therefore cannot rule out differential recall bias potentially leading to an overestimation of the pooled risk estimates. Only one study [17] was based on a job-exposure matrix making differential information bias unlikely.

## Conclusions

Due to high heterogeneity of exposure assessment in the available studies, earlier meta-analyses were not able to determine the dose-response relationship between manual handling of loads at work and hip osteoarthritis risk. In a newly developed meta-regression approach, we made use of a reference population to uniformly replace the exposure categories of the available primary studies with cumulative exposure values. Using this methodological approach, we were able to estimate the exposure to lifting and/or carrying of loads which would result in a “doubling risk” of hip osteoarthritis for men (the “doubling dose”). Due to methodological limitations, the derived doubling dose values are subject to large uncertainties. As best estimates, we found doubling doses between about 6000 and 14,000 cumulative tons of weights ≥20 kg handled, between 6000 and 10,500 cumulative tons of weights ≥20 kg handled > 10 times/day, and between 218,000 and 514,000 cumulative lifting and/or carrying operations of weights ≥20 kg for men. Assuming a working life of 40 years and a working-year comprised of 220 days, the workload needed to achieve the doubling risk would be equivalent to either performing between 25 to 58 lifting and/or carrying operations of weights ≥20 kg or lifting 0.7 to 1.6 tons per day. In workplaces where these intense physical workload exposure might occur, preventive measures need to be intensified to avoid hip osteoarthritis and other work-related musculoskeletal diseases.

## Additional file

Additional file 1: **Table S1.** Cumulative exposure percentiles of the male reference population restricted to individuals ≥50 years. (DOC 28 kb)

## Abbreviations

appr: Approximately
OA: Hip osteoarthritis
OR: Odds ratio
