# Supplementary material for: Dose-response relationship between cumulative physical workload and osteoarthritis of the hip – a meta-analysis applying an external reference population for exposure assignment
Source: BMC Musculoskelet Disord. 2018 Jun 1;19:182. doi: 10.1186/s12891-018-2085-8 (PMC5984732; doi:10.1186/s12891-018-2085-8)
Supplement: Supplementary file 1 — Table S1. Cumulative exposure percentiles of the male reference population restricted to individuals ≥50 years. (DOC 28 kb) [file 12891_2018_2085_MOESM1_ESM.doc]

**Online Supplement**

**Table S1**: Cumulative exposure percentiles of the male reference population restricted to individuals ≥50 years

| **Exposure parameter** | **10^th^** | **20^th^** | **30^th^** | **40^th^** | **50^th^** | **60^th^** | **70^th^** | **80^th^** | **90^th^** | **100^th^** |
| --- | --- | --- | --- | --- | --- | --- | --- | --- | --- | --- |
| a. cumulative tons of weights ≥20 kg | 0 | 0 | 0 | 8 | 53 | 493 | 1,226 | 3,003 | 7,177 | 307,813 |
| b. cumulative tons of weights ≥20 kg handled >10 times/day | 0 | 0 | 0 | 0 | 0 | 132 | 968 | 2,620 | 6,596 | 307,813 |
| c. cumulative number [x 1,000] of lifting and/or carrying operations of weights ≥20 kg | 0 | 0 | 0 | 0.3 | 1.8 | 13 | 41 | 88 | 280 | 13,463 |
